# Risk Factors for Anthracycline-Induced Cardiotoxicity in Breast Cancer Treatment: A Meta-Analysis

**DOI:** 10.3389/fonc.2022.899782

**Published:** 2022-06-17

**Authors:** Meilin Zhang, Hongguang Yang, Changcun Xu, Feng Jin, Ang Zheng

**Affiliations:** ^1^ Department of Burn Plastic Surgery, Chaoyang Central Hospital, Chaoyang, China; ^2^ Department of Cardiology, Chaoyang Central Hospital, Chaoyang, China; ^3^ Department of Breast Surgery, The First Affiliated Hospital of China Medical University, Shenyang, China

**Keywords:** breast cancer, anthracyclines, cardiotoxicity, meta-analysis, risk factors

## Abstract

**Background:**

Anthracyclines play an important role in the treatment of breast cancer (BC) and other malignant tumors. However, accompanied side-effects are non-ignorable. The purpose of this meta-analysis is to determine the risk factors for anthracycline-induced cardiotoxicity (ACT), so as to identify high-risk patients.

**Methods:**

The search for literature was conducted in PubMed, The Cochrane Library, Embase and Web of science. Records were selected with inclusion criteria and exclusion criteria. The newcastle-ottawa scale (NOS) was used to assess the quality of literature, and Review Manager 5.3 software was used for meta-analysis.

**Results:**

Thirteen studies met the inclusion criteria. Meta-analysis indicated that risk factors for ACT were use of trastuzumab (odds ratio [OR]: 2.84, 95% confidence interval [CI]: 2.49-3.22, *p <* 0.00001), cumulative dose of anthracyclines (OR: 1.45, 95%CI: 1.28-1.65, *p <* 0.00001), hypertension (OR: 2.95, 95%CI: 1.75-4.97, *p <* 0.0001), diabetes mellitus (DM) (OR: 1.39, 95%CI: 1.20-1.61, *p <* 0.0001), tumor metastasis (OR: 1.91, 95%CI: 1.17-3.11, *p =* 0.009) and coronary heart disease (CAD) (OR: 2.17, 95%CI: 1.50-3.15, *p <* 0.0001). In addition, our analysis revealed that body mass index (BMI) had no effect on ACT (OR: 1.18, 95%CI: 0.98-1.43, *p =* 0.08).

**Conclusions:**

Patients with high risk for ACT can be identified by these factors. For such patients, a higher level of monitoring and protection for the cardiac function should be performed by clinicians.

**Systematic Review Registration:**

INPLASY, identifier INPLASY202250140.

## Introduction

Anthracyclines play an important role, even when targeted therapy and immunotherapy are emerging. Anthracyclines are broad-spectrum, effective and widely used in the treatment for solid tumors and hematological malignancies, including breast cancer (BC), gastric cancer (1), ovarian cancer (2), leukemia (3) and lymphoma (4). At present, in the field of BC treatment, commonly used anthracyclines include: doxorubicin, epirubicin and so on. However, despite its outstanding contribution to anti-tumor therapy, concomitant side-effects are non-ignorable, including hair loss, bone marrow suppression, gastrointestinal reactions and cardiotoxicity. The most serious one of which is cardiotoxicity. It can produce arrhythmia, heart failure, hypertrophic cardiomyopathy and other cardiac adverse events, which seriously reduces the quality of patients’ life and endangers their health. Cardiotoxicity, as the most serious adverse reaction of anthracyclines, has no unified definition currently. Generally, cardiotoxicity is defined as >10% decrease in left ventricular ejection fraction (LVEF) from baseline and LVEF <50% on multigated acquisition or LVEF <55% on echocardiography (5, 6). In China, according to the 2021 Guidelines of Chinese Society of Clinical Oncology (CSCO), anthracycline cardiovascular toxicity is defined as: heart failure and coronary artery disease (3%-48%), bradycardia, sinus tachycardia, atrioventricular block, atrial fibrillation, supraventricular tachycardia, ventricular tachycardia/fibrillation and acute myocarditis (7). Among BC survivors, the primary cause of death unrelated to cancer diagnosis is cardiovascular disease, while chemotherapy is closely related to the long-term cardiotoxicity for BC patients (8). The research on ACT is helpful to improve the prognosis of BC patients. At present, the incidence of ACT is about 5% (9). With the increase of the number of people using anthracyclines, more attention to cardiotoxic events should be paid. However, based on the current detections, it seems impossible to achieve precise identification for this group. In the treatment of BC, ACT is usually progressive and irreversible, which can cause heart damage at the first use and accumulate, thus affecting the continuation of chemotherapy. Therefore, early identification of high-risk patients with cardiotoxicity is particularly indispensable, and effective prevention during chemotherapy is a wise choice. In addition, some scholars have devoted themselves to the exploration for risk factors, but no consensus has been reached yet. From the research at home and abroad, the results of different studies were not consistent (10–12). To our knowledge, the study is one of the few meta-analyses of risk factors for ACT. By analyzing these risk factors, clinicians can administer cardioprotective measures in time and conduct heart monitoring during the treatment, ultimately reducing the occurrence of ACT and ensuring a better prognosis for BC patients.

## Methods

### Search Strategy

This meta-analysis was performed according to the Preferred Reporting Items for Systematic Reviews and Meta-analyses (PRISMA) (13). The search for literature was conducted in PubMed, The Cochrane Library, Embase and Web of Science. The search strategy could be divided in three steps: Firstly, the type of disease was retrieved by the combination of subject words and free words, and “OR” was used in the middle. Cardiotoxicity, anthracyclines and breast neoplasms were linked by “AND”. Secondly, the retrieval of research objectives was carried out. The retrieval of etiology was referred to McMaster university. “AND” was used between the first step and the second step. Finally, cardiotoxicity, anthracyclines, breast neoplasms, risk factors and their free words were selected as the retrieval words. It was searched independently by two researchers, and reached a consensus through discussion when the results diverged.

### Selection of the Studies

Inclusion criteria: (1) The source of the case was BC patients diagnosed by medical institutions and received adjuvant treatment with anthracyclines. (2) The type of design was a case-control study or cohort study. (3) Relevant studies on the influencing factors for ACT were included (influencing factors for ACT were mentioned in at least three literature). (4) Odds ratio (OR)/hazard ratio (HR) and 95% confidence interval (CI) were provided in the results, or sufficient data could be provided for calculation.

Exclusion criteria: (1) Repeated publication of literature. (2) Unable to obtain the full text, incomplete data, or incorrect statistical methods. (3) Reviews, meta-analyses, conferences, comments, case reports and animal experiments. (4) Newcastle-ottawa scale (NOS) score < 6. (5) The definition of risk factors was significantly different from general standards or most studies.

### Data Extraction and Quality Assessment

After searching the literature in the database, we firstly excluded repetitive literature. Secondly, we excluded outmoded literature published before the year of 2000. Then we eliminated the meta-analyses, reviews, case reports, meetings, comments and animal experiments. Next, we deleted the articles that did not conform to the research by reading the title and abstract. Finally, we downloaded the full texts of left-behind studies, and retained the clinical studies that conformed the intention and inclusion criteria.

Two researchers extracted the relevant data from the included literature: author, year of publication, country, type of study, number of patients and risk factors for ACT (mentioned in at least three articles).

This Meta-analysis used the NOS recommended by the Cochrane Collaboration for quality assessment. The NOS is mostly used in cohort studies or case control studies and includes selection, comparability and outcome/exposure, with a total eight items. A study can be awarded a maximum of one star for each numbered item within the selection and outcome/exposure categories. A maximum of two stars can be given for comparability. Stars range from zero to nine, and six stars and above are considered high-quality literature (14). The NOS was done by two researchers independently, and the third researcher will resolve them if the results are different.

### Data Processing

Review Manager 5.3 software was used for data analysis. Cochrane Q test and I^2^ were used to analyze the heterogeneity among studies. When *p* > 0.1 and *I^2^* < 50%, it indicated that there was no statistical heterogeneity and fixed effect model was used for data analysis. If there was statistical heterogeneity and the degree of heterogeneity was acceptable, we would choose the random effect model for analysis. Otherwise, we should conduct a sensitivity analysis to evaluate the source of heterogeneity. After excluding the literature that had an obvious effect on heterogeneity, if heterogeneity did not exist, we would continue to use the fixed effect model for meta-analysis. The final results were shown by forest plot, and the publication bias was analyzed by funnel plot.

## Results

**Figure 1:**
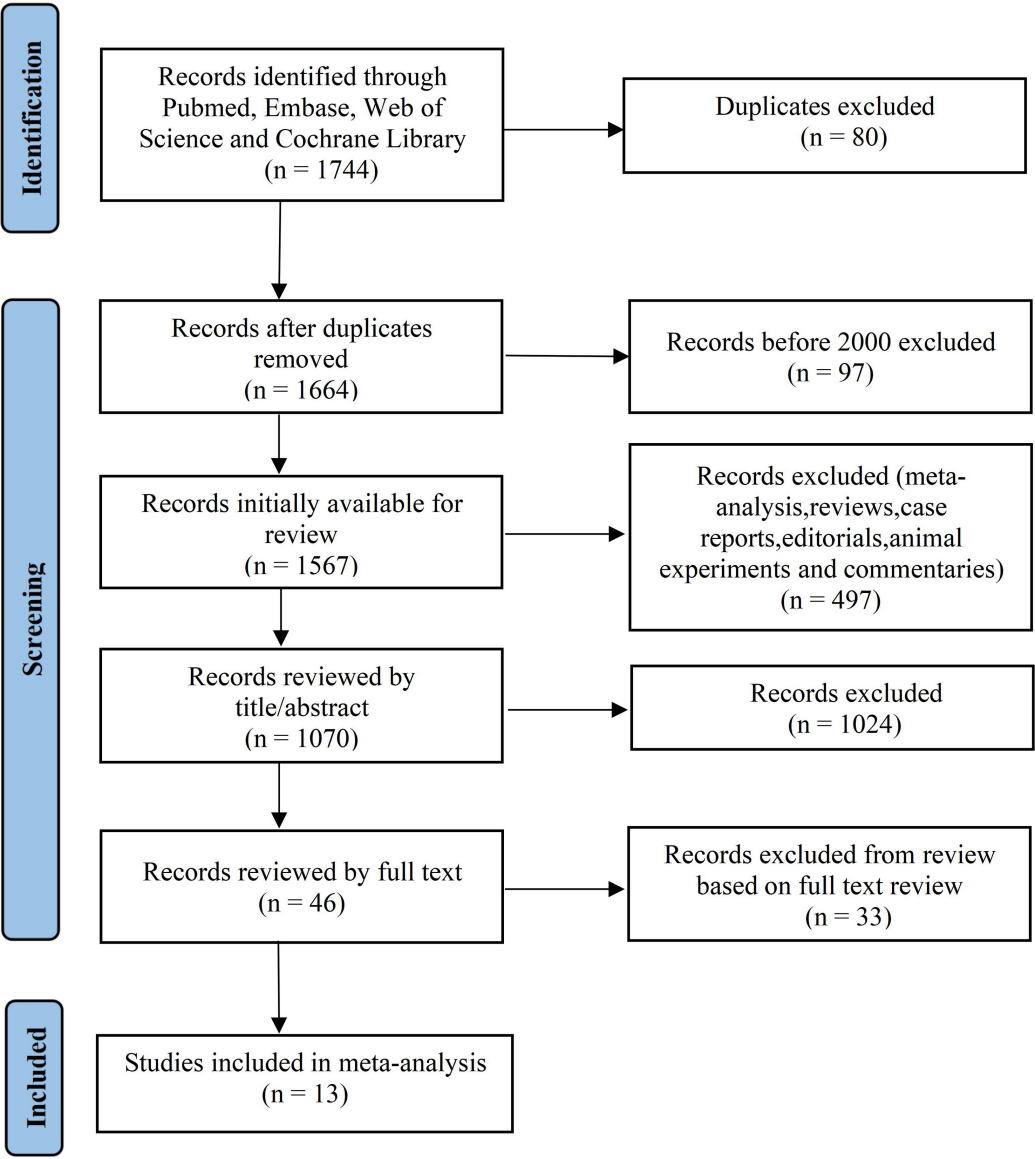
Flowchart review design.

We selected 364, 734, 41, and 605 studies from PubMed, The Cochrane Library, Embase and Web of science, with a total of 1744. Among them, 80 duplicated studies and 97 (before 2000) were deleted. After the screening of 1521 irrelevant records, the remaining 46 were read in full text and carefully evaluated. Finally, 13 articles (15–27) were included in the final analysis (**Figure 1**) . A total of 56,085 patients were included in the 13 studies, all in English, published between 2008 and 2021. Among them, 2544 cases in the case group (patients with adverse cardiac events after receiving anthracyclines), that is, 4.54% of the BC patients experienced cardiotoxicity. There were 53541 cases in the control group (received anthracyclines but did not have adverse cardiac events, most of the controls were from the hospital) (**Table 1**). The NOS scores varied from 7 to 9, indicating that the quality of the studies was high (**Table 2**). The results showed that Trastuzumab use, hypertension, diabetes mellitus (DM), coronary heart disease (CAD), metastasis and cumulative anthracyclines dose were risk factors for ACT (*p* < 0.05), while BMI was not related to ACT (*p* = 0.08) (**Table 3**).

**Table 1:** Characteristics of the studies and population in the included articles.

Study	Country	NO. of centers	Type of study	Population	NO. of subjects	Case	Control	Treatment	Cardiotoxicity definition	Risk factors
Jukapun 2021 (15)	Thailand	Single center	Retrospective	BC (any stage)	475	15	460	Anthracycline-based with or without trastuzumab	A decrease in LVEF >10% from baseline to <50%	1.
Boram 2020 (16)	Korea	Single center	Retrospective	EBC	257	42	215	Neoadjuvant/adjuvant doxorubicin therapy with or without trastuzumab	A >10% reduction in the LVEF from baseline and LVEF < 50% on MUGA, or <55% on echocardiography	1.2.
Domas 2020 (17)	Lithuanian	Single center	Prospective	BC stageI-III	73	17	56	Doxorubicin-based chemotherapy	A decrease of LVEF by more than 10% after chemotherapy	3.
Hyunsoon 2020 (18)	Korea	Single center	Retrospective	BC	613	92	521	Doxorubicin chemotherapy	> 10 percentage points reduction in LVEF from baseline and LVEF < 50% on MUGA or < 55% on echocardiography; > 15 percentage point reduction in LVEF from baseline with LVEF > 50% on MUGA or > 15 percentage point reduction in LVEF from baseline with LVEF > 55% on echocardiography	1.6.
Elise 2019 (19)	French	Multi-center	Prospective	Non-metastatic invasive BC (cT0 to cT3, CN0–3)	929	29	900	Anthracycline and/or trastuzumab chemotherapy	A reduction in LVEF > 10 percentage points from baseline to LVEF < 50%	2.3.
György 2019 (20)	Hungarian	Multi-center	Retrospective	EBC	8068	557	7511	Epirubicin adjuvant treatment	I50 International Classification of Diseases-10 code	3.4.5.6.7.
Young 2018 (21)	Korea	Multi-center	Retrospective	BC	43586	1482	42104	Doxorubicin adjuvant treatment	International Statistical Classification of Diseases and Related Health Problems 10th Revision (ICD‐10)	1.2.4.5.6.
Edward 2017 (22)	USA	Single center	Retrospective	BC	411	21	390	Anthracycline-based adjuvant/neoadjuvant chemotherapy	Clinical CHF with ejection fraction below 50 percent or an asymptomatic decline in ejection fraction by 10% or more to below 50 percent	1.3.
Paul 2016 (23)	England	Multi-center	Retrospective	EBC	165	34	131	Anthracyclines and/or trastuzumab chemotherapy	A subclinical fall in LVEF ≥10% to below normal	1.3.7.
Raquel 2015 (24)	Columbus	Multi-center	Retrospective	BC	162	52	110	Adriamycin and cytoxan chemotherapy	A drop in ejection fraction to <50 % or >15 % decrease from pre-therapeutic levels, and those who developed a new arrhythmia or myocardial infarction after therapy	1.4.
JOSÉM 2015 (25)	Spain	Single center	Prospective	BC	85	49	36	Anthracycline-based with or without trastuzumab	New-onset heart failure according to Framingham criteria;symptomatic decline ≥ 5%, or asymptomatic decline ≥10% to an LVEF < 55%; onset of sustained ventricular tachycardia; sudden cardiac death	2.
Woo-Baek 2013 (26)	Korea	Multi-center	Retrospective	BC (18-65years old)	174	29	145	Doxorubicin-containing chemotherapeutic	The LVEF decreases more than 10% from the baseline or the LVEF declines under 55%	1.5.7.
Marianne 2008 (27)	Danish	Single center	Retrospective	Metastatic BC	1087	125	962	Epirubicin-based chemotherapy	Subjective and objective signs of CHF in combination with either a chest x-ray revealing cardiomegaly with or without pulmonary congestion or pleural effusion	7.

BC, breast cancer; EBC, early breast cancer; LVEF, left ventricular ejection fraction; CHF, congestive heart failure; MUGA, multigated acquisition.

1. trastuzumab use 2. body mass index 3. hypertension 4. diabetes mellitus.

5. coronary heart disease 6. metastasis 7. cumulative anthracyclines dose.

**Table 2:** Literature quality evaluation: Newcastle-Ottawa Scale.

Study	Selection	Comparability	Outcome/Exposure	NOS score
Jukapun 2021	☆☆☆☆	☆☆	☆☆☆	9
Boram 2020	☆☆☆	☆☆	☆☆	7
Domas 2020	☆☆☆	☆☆	☆☆	7
Hyunsoon 2020	☆☆☆	☆☆	☆☆	7
Elise 2019	☆☆☆	☆☆	☆☆☆	8
György 2019	☆☆☆	☆☆	☆☆	7
Young 2018	☆☆☆	☆☆	☆☆	7
Edward 2017	☆☆☆	☆☆	☆☆☆	8
Paul 2016	☆☆☆	☆☆	☆☆☆	8
Raquel 2015	☆☆☆☆	☆☆	☆☆☆	9
JOSÉM 2015	☆☆☆	☆☆	☆☆	7
Woo-Baek 2013	☆☆☆	☆☆	☆☆	7
Marianne 2008	☆☆☆	☆☆	☆☆	7

NOS, Newcastle-Ottawa Scale.

☆: A star represents a score.

**Table 3:** Meta-analysis for ACT risk factors.

Risk factors	No.of studies	OR	95%CI	Heterogeneity test		Overall effect test	
				*I²*(%)	*p* value	*z* value	*p* value
Trastuzumab use	7	2.84	2.49-3.22	39	0.13	15.92	<0.00001
BMI	3	1.18	0.98-1.43	77	0.01	1.74	0.08
Hypertension	5	2.95	1.75-4.97	86	<0.0001	4.06	<0.0001
DM	3	1.39	1.20-1.61	42	0.18	4.34	<0.0001
CAD	3	2.17	1.50-3.15	51	0.13	4.11	<0.0001
Metastasis	3	1.91	1.17-3.11	89	0.0002	2.60	0.009
Cumulative anthracyclines dose	3	1.45	1.28-1.65	0	0.54	5.71	<0.00001

BMI, body mass index; DM, diabetes mellitus; CAD, coronary heart disease; OR, odds ratio; CI, confidence interval; I^2^, Cochran Q test.

### Trastuzumab Use

**Figure 2:**
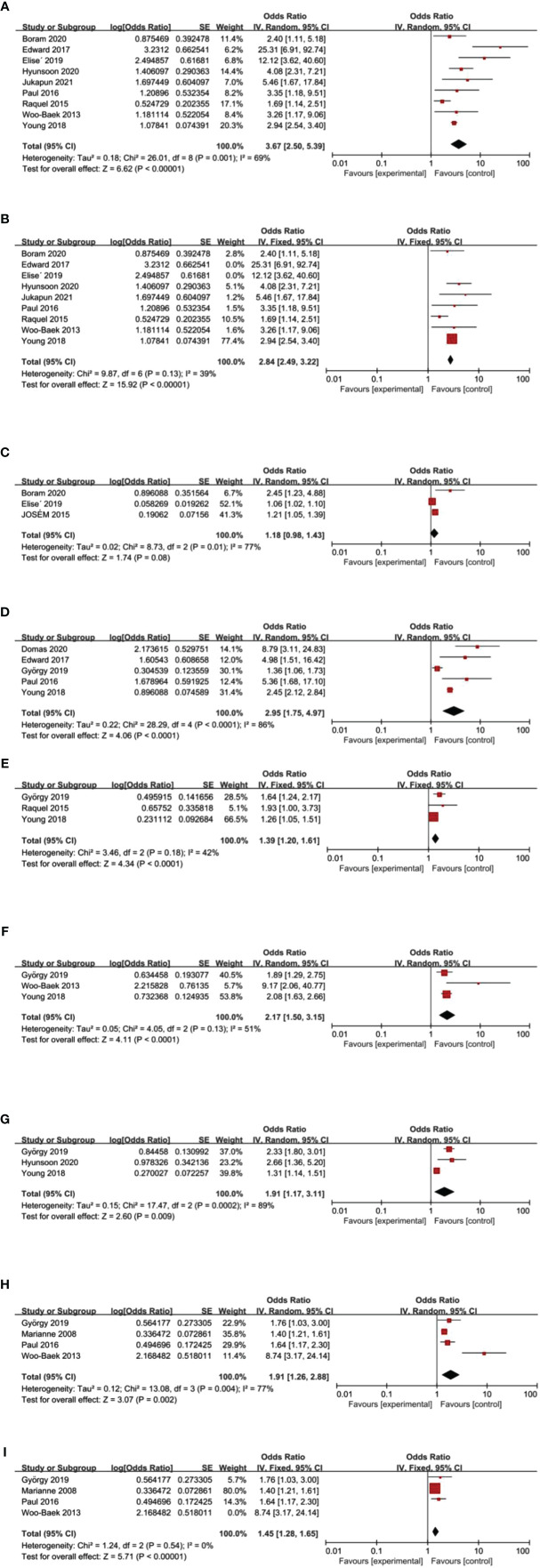
Forest plot results for risk factors. **(A)** trastuzumab use: before sensitivity analysis. **(B)** trastuzumab use: after sensitivity analysis. **(C)** BMI **(D)** hypertension **(E)** DM **(F)** CAD. **(G)** metastasis **(H)** cumulative anthracyclines dose: before sensitivity analysis. **(I)** cumulative anthracyclines dose: after sensitivity analysis.

A total of nine articles (15, 16, 18, 19, 21–24, 26) studied the relationship between Trastuzumab use and ACT. After meta-analysis, the results were shown in **Figure 2A**. It could be clearly seen that the results were heterogeneous (*I^2^* = 69%, *p* = 0.001). Therefore, we continued the sensitivity analysis and found that Edward 2017 and Elise ‘ 2019 were the main causes of heterogeneity. After deletion, meta-analysis was performed again, and the results were shown in **Figure 2B**. There was no heterogeneity after the deletion of two articles (*I^2^* = 39%, *p* = 0.13). A fixed effect model was selected for meta-analysis. It was concluded that trastuzumab use was a risk factor for ACT (OR: 2.84, 95%CI: 2.49-3.22, *p <* 0.00001). The risk of ACT among BC patients who use trastuzumab was 2.84 times higher than those who did not use trastuzumab.

### BMI

Based on the data of three articles (16, 19, 25), the results of BMI were shown in **Figure 2C** (*I^2^* = 77%, *p* = 0.01). Therefore, a random effect model was selected for analysis and the result showed that BMI was not a risk factor for ACT (OR: 1.18, 95%CI: 0.98-1.43, *p =* 0.08).

### Hypertension

We chose a random effect model for hypertension (*I^2^* = 86%, *p* < 0.1). The analysis of data from five studies (17, 20–23) pointed out that hypertension was associated with ACT (OR: 2.95, 95%CI: 1.75-4.97, *p <* 0.0001). Compared with people who did not suffer from hypertension, BC patients with hypertension were more likely to develop ACT (**Figure 2D**).

### DM

Three articles (20, 21, 24) have studied the relationship between DM and ACT. The result was not heterogeneous (*I²* = 42%, *p* = 0.18). So we used the fixed effect model for meta-analysis. The result revealed that DM was an influence factor for ACT (OR: 1.39, 95%CI: 1.20-1.61, *p <* 0.0001). We found that BC patients with DM were 1.39 times more likely to develop ACT than those who without DM (**Figure 2E**).

### CAD

The association between CAD and the development of ACT was examined in three studies (20, 21, 26). The result was slightly heterogeneous (*I²* = 51%, *p* = 0.13), and the random effect model was chosen. Finally, CAD was a risk factor for ACT in our results (OR: 2.17, 95%CI: 1.50-3.15, *p <* 0.0001) (**Figure 2F**).

### Metastasis

Tumor metastasis was found to be a risk factor for ACT, based on the analysis of three studies (18, 20, 21) (OR: 1.91, 95%CI: 1.17-3.11, *I^2^* = 89%, *p=*0.009) (**Figure 2G**).

### Cumulative Anthracyclines Dose

Four articles (20, 23, 26, 27) studied the relationship between the cumulative anthracyclines dose and ACT. Three of them (20, 23, 26) did not reach the maximum cumulative dose of anthracyclines. Some patients with epirubicin met or exceeded the maximum cumulative dose in only one study (27) (maximum cumulative dose of anthracyclines based on the CSCO guidelines). After meta-analysis, heterogeneity was obvious (*I²*=77%, *p* = 0.004) (**Figure 2H**).We found Woo-Baek 2013 was the main cause of heterogeneity. There was no heterogeneity after deleting Woo-Baek 2103 (*I²*= 0%, *p* = 0.54) (**Figure 2I**). Finally, it was concluded that cumulative anthracyclines dose was a risk factor for ACT (OR: 1.45, 95%CI: 1.28-1.65, *p <* 0.00001).

**Figure 3:**
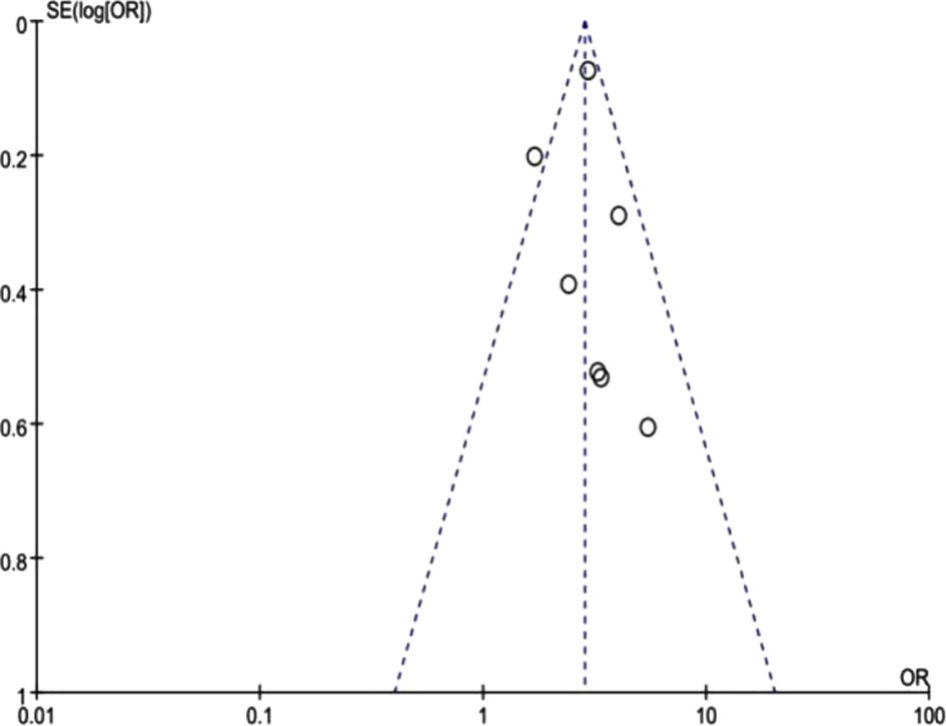
Funnel plot of trastuzumab use.

As the number of articles included in the above factors was limited, we took the funnel plot of trastuzumab as an example. The seven studies (15, 16, 18, 21, 23, 24, 26) were basically located within the 95%CI and symmetrically distributed, suggesting no obvious publication bias (**Figure 3**).

## Discussion

Anthracyclines play an irreplaceable role in the BC treatment. Cardiotoxicity, as the most serious side-effect of anthracyclines, is of concern to clinicians for its mechanism, risk factors, monitoring and prognosis. In terms of mechanism, some conclusions have been achieved with doubts remaining. It was generally believed that cardiotoxicity was related to the damage of oxygen free radicals and TOP II (28, 29).

We urgently need to explore the risk predictors for ACT to identify high-risk patients. In this study, related studies from 2000 to 2021 were collected, and risk factors for ACT were analyzed comprehensively. The results of meta-analysis showed that trastuzumab use, cumulative dose of anthracyclines, hypertension, DM, tumor metastasis and CAD predicted a higher risk for ACT independently. In addition, we found that some risk factors in the analysis had varying degrees of heterogeneity. Statistical heterogeneity may be caused by differences in research objects or statistical methods. Besides, some risk factors were analyzed in the limited literature. These reasons might result in the potential bias and statistical heterogeneity. In the results, we found that the OR of hypertension was the highest. However, some literature suggested that hypertension was not an independent influencing factor for ACT in multivariate analysis (18, 19). DM was one of the conventional cardiovascular risk factors. We found that BC patients with DM were more likely to develop ACT. But Domas et al. (17) showed that DM was not associated with left ventricular systolic dysfunction caused by doxorubicin. Hence, further investigations were warranted. BMI was not demonstrated a risk factor for ACT in this study. On the contrary, Maaroufi et al. (30) followed 473 patients treated with anthracyclines and trastuzumab, including 90 for obesity (BMI ≥ 30kg/m^2^) and 146 for overweight (25kg/m^2^ ≤ BMI ≤ 29.9kg/m^2^) and concluded that obesity was significantly associated with a higher risk of cardiotoxicity by multivariate analysis. Likewise, Charles et al. (31) included 15 studies and 8,745 patients with BC who were treated with anthracyclines, indicating that obesity and overweight were risk factors for ACT. Another study included 967 human epidermal growth factor receptor 2 (HER2)-negative BC patients treated with adriamycin. Multivariate analysis pointed out that BMI was relevant to ACT (10). Similarly, some studies have found that higher BMI was related to ACT in the treatment of BC (32–35). It can clearly be seen that this study did not get consistent results with the above. The reason may be that only three articles were included in this meta-analysis, which resulted in bias and affected the results. Therefore, the relationship between BMI and ACT needs more adequate research in the future. In this study, we revealed that the use of trastuzumab increased the risk for ACT. Trastuzumab is used in adjuvant therapy of HER2-positive patients. As we all know, the use of trastuzumab can cause trastuzumab-induced cardiotoxicity (TIC) in patients. Some studies have also explored the influencing factors for TIC. Farolfi et al. studied 179 patients in early-stage BC treated with trastuzumab and found that epirubicin >500 mg/m^2^ or cumulative dose of doxorubicin >240 mg/m^2^ were risk factors for TIC, while TIC was not associated with other cardiac related factors (36). A similar finding was confirmed in the study by Eiger et al., they revealed that cumulative dose of doxorubicin ≥240 mg/m^2^ and of epirubicin ≥480 mg/m^2^ caused cardiac events in HER2-positive BC patients receiving adjuvant lapatinib and/or trastuzumab (37). Besides, in the study by Xue et al., they included 415 patients diagnosed with early BC. Multivariate analysis suggested that CAD, the use of anthracyclines for more than four cycles and radiotherapy exposure were risk factors for TIC (38). Mariana et al. showed that anthracycline use, a more recent year of diagnosis,physician graduating after 1990 and female prescribing physician were factors associated with optimal cardiac monitoring in a large population-based study of older patients with BC (39). From the above results, we can see that the prevention of TIC is equally essential, especially the combination of trastuzumab and anthracyclines. In a randomized trial, both lisinopril and carvedilol were effective in preventing cardiotoxicity in HER2-positive BC patients treated with trastuzumab when concurrently receiving anthracyclines (40). We also found that the cumulative dose of anthracyclines was related to cardiotoxicity. Therefore, limiting the cumulative dose of anthracyclines could effectively reduce the occurrence of cardiotoxicity. Additionally, BC patients with tumor metastasis are high-risk groups for ACT. We hypothesized that tumor metastasis was positively associated with ACT. Unfortunately, the specific mechanism of the relationship between ACT and tumor metastasis remains unclear. After analyzing the reasons, it may be that patients with metastasis have a larger tumor burden, so the use of chemotherapeutic drugs is high-dose, which is easy to aggravate the cardiotoxicity of patients. Combination regimens are generally used in BC patients with metastatic, drug synergy may lead to more serious cardiac adverse events during their treatment course. Moreover, considering the patient’s own conditions, BC patients with metastasis usually have poor tolerance and nutritional status. Therefore, we must formulate individualized treatment regimens for patients and actively take preventive measures during chemotherapy. Among the thirteen included studies, four studies (18, 20, 21, 25) mentioned the influence of age on ACT, with the results supporting age as an independent risk factor for ACT. However, the meta-analysis for age was not performed in this study due to the inconsistencies in thresholds for “young” and “old” across different studies. In the future, we can make a stratified study on age and discuss the relationship between patients of different ages and ACT, which is helpful for clinicians to diagnose and treat them more accurately. In addition, the relationship between radiotherapy and ACT cannot be ignored. There is an obvious dose-effect relationship between the dose of radiation and cardiovascular disease in the literature (41). High-dose radiation therapy (radiation doses ≥30 Gy) increases the incidence of cardiac dysfunction, but current evidence suggests that radiation doses <30 Gy do not cause significant cardiac dysfunction (42, 43). Christof et al. (11) showed that radiotherapy for left-sided breast cancer (radiation dose not mentioned) was an independent risk factor for epirubicin-induced cardiac toxicity by multivariate analysis, that is, radiotherapy increased the risk for ACT. The application of new techniques to decrease the dose of radiation may reduce the incidence of adverse cardiac events. Yoodee et al. (15) included 475 BC patients receiving anthracyclines with or without trastuzumab, and multivariate analysis demonstrated that radiotherapy (radiation dose not mentioned) increased the risk of heart failure in BC patients. Due to the limited number of literature, radiotherapy is not included in this meta-analysis, but this does not mean that we can ignore the effect of radiotherapy on ACT. Except for this, in a prospective study, Abdallah et al. (44) found that BC patients with hyperlipidemia developed left ventricular diastolic dysfunction after chemotherapy. However, Maaroufi et al. (30) obtained the opposite conclusion in their study. Although the incidence of ACT in the dyslipidemia group was higher than that in the patients without dyslipidemia, there was no statistical significant relationship between dyslipidemia and ACT. Therefore, the effect of blood lipids on ACT needs to be confirmed by more studies. The analysis of the effects of drinking (24) and smoking (44) on ACT is done by other researchers but were not included in this meta-analysis due to limited data.

Effective monitoring of anthracyclines is particularly important in practice. In the clinic, cardiotoxicity monitoring methods include ultrasonic electrocardiogram, radionuclide ventricular imaging, biomarkers, magnetic resonance imaging and endocardial myocardial biopsy. The CSCO guidelines recommend that patients with BC should undergo routine electrocardiogram and echocardiography before receiving anthracyclines, in order to assess whether their cardiac function can tolerate chemotherapy. Relevant literature suggests that echocardiography is currently the best monitoring method for LVEF and can be used as the first choice for monitoring cardiac function throughout treatment (45). In terms of biomarkers, troponin is more sensitive and specific in myocardial injury, and is a potentially effective screening tool (46). In addition, the role of brain natriuretic peptide (BNP) and N-terminal pro-brain natriuretic peptide (NT-proBNP) in the early monitoring of the heart is attracting attention increasingly (47, 48). Therefore, we could use the above methods to detect and diagnose ACT patients early. At present, how to maximize the benefits of patients with cancer is not only a major challenge for oncologists. For the problem of cardiotoxicity caused by cancer treatment, it is necessary to establish a multidisciplinary team, including experts in oncology, cardiology, imaging and other related fields. In principle, multi-disciplinary treatment (MDT) should run through the entire process of anti-tumor therapy for patients, and should be adjusted timely according to the changes in the patient’s condition, so as to maximize the prognosis of the patients and prolong their survival. The discipline of cardiac oncology has been developed to optimize the comprehensive treatment of cancer patients.

Prophylaxis is necessary in patients receiving anthracyclines, and the CSCO guidelines recommend the use of dextrazoxane (DRZ) before initial use of anthracyclines to effectively prevent the ACT. DRZ is also the only cardioprotective agent approved by Food and Drug Administration for anthracyclines. In addition, a meta-analysis showed that prophylactic use of angiotension converting enzyme inhibitors (ACEI) could also reduce the clinical or subclinical cardiotoxicity, thus improving the survival of BC patients (49). Wittayanukorn and other scholars got similar results (50). In a multicenter randomised trial, the International CardioOncology Society-one trial (ICOS-ONE) was designed to compare two strategies for enalapril guiding prevention of ACT. All patients received enalapril before chemotherapy was defined as the prevention arm, and enalapril was used only in patients with an increase in troponin during or after chemotherapy was defined as the troponin-triggered arm. There was no statistically significant difference in the treatment strategies between the two groups. The authors considered that a troponin-triggered strategy could be more convenient during their treatment course (51). In addition, the ICOS-ONE trial showed that there was no significant difference in the concentration of biomarkers (troponin I, BNP and pentraxin 3) between the prevention arm and troponin-triggered arm in patients without pre-existing cardiac disease receiving initial chemotherapy at 36 months of follow-up. It does not seem to result in clinically significant cardiac injury in these patients (52). Other cardioprotective agents incorporate coenzyme Q10, n-acetylcysteine, antioxidants and iron chelators, whose protective effect on cardiotoxicity is unclear and needs more research (53, 54). Finally, liposomal anthracyclines, such as liposomal doxorubicin and liposomal daunorubicin may reduce the incidence of ACT and can be considered as an alternative to traditional anthracyclines in patients with clinically known cardiac dysfunction.

We identified the risk factors for ACT, whether we could consider using the model of score to assess the cardiotoxicity of these patients and develop a more comprehensive treatment before adjuvant chemotherapy or neoadjuvant chemotherapy. However, the specific weight of each risk factor is unknown. We are able to construct an ACT prediction model based on the selected risk factors, which is conducive to making clinical decisions. In addition, based on these risk factors, we can identify high-risk patients with ACT, fully assess the patient’s condition, and formulate an individualized treatment plan for BC patients. For high-risk patients of ACT, cardiac function should be monitored and followed up early to avoid the interruption of chemotherapy due to cardiotoxicity and other reasons, so as to ensure the continuity and safety throughout treatment. Regular follow-up is also necessary for BC patients who have completed all treatment. The literature suggests that ACT may occur within one year after chemotherapy, and early detection and aggressive treatment can achieve better efficacy (55).

Although this meta-analysis was carried out strictly conforming with the PRISMA, there were some limitations. In this study, due to the strict inclusion and exclusion criteria, the number of articles included was limited, leading to the unavoidable heterogeneity in our results. Some risk factors could not be analyzed for publication bias. Hence, it was suggested that multicenter and prospective studies should be carried out in the future to provide more scientific evidence for taking targeted measures.

## Conclusion

This meta-analysis showed that trastuzumab use, cumulative dose of anthracyclines, hypertension, DM, tumor metastasis, and CAD were risk factors for ACT. High-risk patients are identified easily in the clinic by these risk factors. We can formulate more precise and individualized treatments for BC patients at different clinical stage who need chemotherapy. It is crucial to administer cardioprotective measures in time and conduct heart monitoring during the treatment, with the purpose of reducing the occurrence of ACT and ensuring a better prognosis for BC survivors.

## Data Availability Statement

The original contributions presented in the study are included in the article/supplementary material. Further inquiries can be directed to the corresponding authors.

## Author Contributions

FJ and AZ were the directors for the fund and conceived this study. MZ collected medical records and drafted manuscript. HY and CX assisted in revising the manuscript. All authors contributed to the article and approved the submitted version.

## Funding

This project was supported by the National Natural Science Foundation of China (82073282), Natural Science Foundation of Liaoning Province (2021-BS115) and China Postdoctoral Science Foundation (2020M681018).

## Conflict of Interest

The authors declare that the research was conducted in the absence of any commercial or financial relationships that could be construed as a potential conflict of interest.

## Publisher’s Note

All claims expressed in this article are solely those of the authors and do not necessarily represent those of their affiliated organizations, or those of the publisher, the editors and the reviewers. Any product that may be evaluated in this article, or claim that may be made by its manufacturer, is not guaranteed or endorsed by the publisher.
